# The domino effect and emotional dysregulation in neurosurgeons

**DOI:** 10.12669/pjms.40.12(PINS).11130

**Published:** 2024-12

**Authors:** Shahar Bano Fatima

**Affiliations:** Dr. Shahar Bano Fatima Post Graduate Resident Neurosurgery, Punjab Institute of Neurosciences, Lahore. Email: shehribani@gmail.com

I am addressing a significant yet frequently overlooked issue in the field of neurosurgery. Pakistan boasts a sizable population, and neurosurgery is one of the most demanding and high-pressure specialties here. According to the World Federation of Neurosurgical Societies (WFNS), an optimal neurosurgery workforce density should be at least 1/200,000 people. However, in Pakistan, the reported density is a mere 0.14/100,000.^1^To meet the minimum requirement, Pakistan needs over four times the current number of neurosurgeons.

Emotional dysregulation occurs when people struggle to manage and control their emotions effectively. Intense and distressing emotions, such as anger, anxiety, or sadness, are inherent parts of the human experience. But when these feelings come too quickly, too often, and are just too difficult to handle, it becomes a concern.^2^

Neurosurgery is a demanding and high-stress specialty involving life-and-death decisions, long hours, intense concentration, and exposure to traumatic events. Although emotional well-being is crucial, the constant pressure and workload often overwhelms neurosurgeons in Pakistan. Mental health issues are often stigmatized and emotional vulnerability is perceived as weakness. The pressure of upholding an image of strength, coupled with the inability to express emotions and a lack of support system, leads to heightened levels of stress and anxiety, making neurosurgeons more prone to emotional dysregulation. This can impair cognitive function and decision-making abilities, potentially compromising patient care and safety.^3^,^4^

Another factor contributing to emotional dysregulation is the relatively low earning potential for neurosurgeons in Pakistan. Unlike in certain other nations where neurosurgeons earn higher salaries, those in Pakistan frequently struggle with financial limitations and reduced compensation despite the long and taxing work hours. This introduces an extra layer of strain and discontent intensifying emotional dysregulation.

It’s essential to acknowledge that emotional dysregulation does not just affect the individual neurosurgeon but the entire team. The effects of emotional dysregulation can snowball, creating a domino effect within the surgical team resulting in misunderstandings, miscommunications, and conflicts that foster a hostile work environment. When one team member lashes out on the junior residents or paramedic staff, it can trigger chain events of similar responses in others, exacerbating the overall stress and tension. This negative cycle can compromise team cohesion, morale, and ultimately, patient outcomes. As a neurosurgery resident, I have witnessed firsthand, the profound impact that emotional stress and burnout can have on the well-being and effectiveness of neurosurgeons and their teams.

Addressing emotional dysregulation in neurosurgeons requires more practical solutions that are attainable within the constraints of Pakistan’s healthcare system. While individual interventions such as counseling and stress management remains valuable, we must also focus on systemic changes that create a supportive work environment. This includes implementing regular team debriefings to discuss challenges. Providing faculty communication training to enhance teamwork and conflict resolution skills. To enhance mental health support, it is essential to establish regular feedback mechanisms. This feedback system can be used to identify areas for improvement. Collaborating with mental health professionals can provide valuable support networks for neurosurgery professionals. Further research to evaluate evolving needs of the community can be conducted. Promoting a culture of openness and reducing stigma around mental health within the institutions can encourage neurosurgeons to seek help when needed.

To address the monetary struggles, there is a pressing need to allocate more funding towards residency programs, ensuring that trainees receive adequate compensation for their work and are not burdened by financial stress. Overcoming these barriers can create an environment that prioritizes the emotional well-being of neurosurgeons and, ultimately improve patient care.
